# The impact of tetrahydrocannabinol on central pain modulation in chronic pain: a randomized clinical comparative study of offset analgesia and conditioned pain modulation in fibromyalgia

**DOI:** 10.1186/s42238-025-00348-x

**Published:** 2025-11-06

**Authors:** Yara Agbaria, Raz Preger, Valerie Aloush, Jacob N. Ablin, Haggai Sharon, Giris Jacob

**Affiliations:** 1 School of Medicine, Gray Faculty of Medical and Health Sciences, Tel Aviv University, Tel Aviv, Israel; 2 J. Recanati Autonomic Research Center, Tel Aviv Sourasky Medical Center, Tel Aviv, Israel; 3 Institute of Pain Medicine, Department of Anesthesiology and Critical Care Medicine, Tel Aviv Sourasky Medical Center, Tel Aviv, Israel; 4 Sagol Brain Institute, Tel Aviv Sourasky Medical Center, Tel Aviv, Israel; 5 Sagol School of Neuroscience, Tel Aviv University, Tel Aviv, Israel

**Keywords:** Tetrahydrocannabinol, Pain relief, Conditioned pain modulation, Offset analgesia, Fibromyalgia, Central sensitization

## Abstract

**Background:**

Tetrahydrocannabinol (THC) has shown efficacy in alleviating chronic pain, particularly in disorders characterized by central sensitization. Offset analgesia (OA) and conditioned pain modulation (CPM) are key biomarkers used to evaluate central pain modulation. This study aimed to compare the effects of THC on OA and CPM in fibromyalgia syndrome (FMS), a prototypical condition of central sensitization.

**Methods:**

In a randomized, double-blind, placebo-controlled crossover design, 23 FMS patients participated in two experimental sessions. Each session included the McGill Pain Questionnaire, visual analogue scale (VAS) assessments, and evaluations of OA and CPM, conducted both before and after sublingual administration of either THC (0.2 mg/kg) or placebo.

**Results:**

THC significantly reduced spontaneous pain ratings on the McGill scale compared to both baseline and placebo (*P* = 0.01 and *P* = 0.02, respectively). THC also significantly enhanced OA relative to baseline and placebo (*P* = 0.04 and *P* = 0.008), while no effect was observed on CPM (*P* = 0.27). Notably, baseline OA magnitude significantly predicted THC-induced pain relief (R² = 0.404, *P* = 0.003), whereas CPM did not show a significant association (*P* = 0.121).

**Conclusions:**

This is the first study to evaluate THC’s distinct effects on central pain modulation using both OA and CPM. THC selectively enhanced OA without influencing CPM, highlighting differential neural mechanisms underlying these paradigms. Furthermore, OA predicted treatment response, suggesting its potential as a biomarker for personalized cannabinoid-based therapies in FMS and other central sensitization disorders.

**Trial registration:**

The study was prospectively registered on ClinicalTrials.gov (ID: NCT05644054) at 1.1.2023. Further details can be found at: https://clinicaltrials.gov/study/NCT05644054?locStr=Israel&country=Israel&cond=fibromyalgi215a%20&intr=THC&aggFilters=status:not%20rec&rank=1***.***

**Supplementary Information:**

The online version contains supplementary material available at 10.1186/s42238-025-00348-x.

## Introduction

Cannabis, particularly its psychoactive component delta-9-tetrahydrocannabinol (THC), has attracted increasing attention as a therapeutic option for chronic pain management (Häuser 2017; Safi et al. 2024). Clinically, THC has been shown to reduce pain intensity (Fitzcharles et al. 2021; Chaves et al. 2020; Blake et al. 2006), improve quality of life (Chaves et al. 2020), and attenuate hyperalgesia in various chronic pain conditions, including neuropathic pain and fibromyalgia (Fitzcharles et al. 2021; Skrabek et al. 2008). However, the specific neural pathways and mechanisms responsible for its analgesic properties, especially in the context of central pain syndromes, require further elucidation.

Abnormalities in central pain inhibition, which normally enable top-down suppression of nociceptive input, play a pivotal role in the persistence of chronic pain (Woolf 2011; Latremoliere and Woolf 2009). Endogenous pain control relies heavily on descending inhibitory pathways originating in the prefrontal cortex (PFC), anterior cingulate cortex (ACC), amygdala, and periaqueductal gray (PAG), which project through brainstem structures such as the rostral ventromedial medulla (RVM) to regulate spinal dorsal horn activity (Huynh 2023; Cifre et al. 2012; Phillips and Clauw 2011; Gracely et al. 2002; Ioachim 2022). In chronic pain conditions, functional and structural alterations in these regions weaken inhibitory processes and shift the balance toward pain facilitation, thereby amplifying nociceptive signaling. This breakdown of endogenous inhibition is a hallmark of central sensitization, a state of heightened excitability within the central nervous system in which normally innocuous or mildly painful stimuli are perceived as strongly painful. Such maladaptive plasticity enhances pain perception and disrupts normal pain modulation (Woolf 2011; Latremoliere and Woolf 2009).

THC is thought to exert its analgesic effects in part by modulating these disrupted pain networks. Specifically, THC interacts with the endocannabinoid system, particularly through CB1 receptors in key pain-modulatory regions of the brain (Nahman-Averbuch and Timmers 2023; Ossipov et al. 2010; Honigman et al. 2013), including the anterior cingulate cortex (ACC), amygdala, and PAG (Alter et al. 2022; Li et al. 2022). To better characterize the neural mechanisms underlying central pain modulation in FMS within this framework, we employed two psychophysical paradigms: conditioned pain modulation (CPM) and offset analgesia (OA). These paradigms are widely used to assess endogenous pain modulation profiles and, although not developed specifically to study THC, they provide a valuable framework for evaluating the effects of THC on pain processing in this context. CPM is a phenomenon whereby the perception of pain at one location is modulated by the application of a noxious stimulus at a different location (Nahman-Averbuch and Timmers 2023; Ossipov et al. 2010; Chakrabarty 2007; Staud et al. 2001). Conversely, OA is defined as a disproportionate decrease in pain intensity after a slight reduction in a noxious temperature, representing a neurobiological mechanism of temporal filtering of pain signals (Cagnie et al. 2014; Julien et al. 2005).

In this study, fibromyalgia syndrome (FMS) was used as a clinical model to explore THC’s modulation of pain pathways associated with central sensitization. Characterized by widespread musculoskeletal pain (Chakrabarty 2007), fatigue, and heightened pain sensitivity, alongside neural changes demonstrating central sensitization(Staud et al. 2001; Cagnie et al. 2014), FMS presents a representative condition for studying disrupted CPM (Julien et al. 2005; Potvin and Marchand 2016)and OA (Oudejans et al. 2015) responses, reflecting impaired pain inhibitory control.

Given that THC has demonstrated beneficial effects on chronic pain -particularly of central origin- and that both CPM and OA represent distinct yet complementary mechanisms of central pain modulation that are disrupted in FMS, we aimed to investigate whether THC differentially enhances CPM, which is primarily mediated by brainstem-level pathways, and OA, which depends more on higher-order cognitive and emotional processes, in FMS patients. We further sought to assess whether these modulatory effects are associated with the clinical analgesic benefits of THC in FMS patients.

## Methods

The study was approved by the Tel Aviv Sourasky Medical Center Research Ethics Committee (Protocol No. TLV-320-016) and conducted in accordance with the principles of the Declaration of Helsinki. Prior to participation, all subjects provided written informed consent after receiving a detailed explanation of the study’s aims and procedures (Protocol Version 3.4, approved on 22.3.2022).

The study was prospectively registered on ClinicalTrials.gov (ID: NCT05644054). Further details can be found at:

https://clinicaltrials.gov/study/NCT05644054?locStr=Israel&country=Israel&cond=fibromyalgi215a%20&intr=THC&aggFilters=status:not%20rec&rank=1 ***.***

This study was conducted as part of a larger clinical trial registered at the link previously provided. In the original trial, a total of 40 participants were recruited. The initial protocol included fMRI scanning as well as OA and CPM testing for all participants. However, after testing the first five participants, who completed both the behavioral assessments and the fMRI scan, it became evident that the length and complexity of the sessions posed significant logistical challenges. Consequently, a power analysis was conducted (see Sect. 2.8 for details) to determine the minimum number of participants required per experimental group (fMRI vs. psychophysical testing). The analysis indicated that at least 17 participants were needed in each group to ensure sufficient statistical power. Accordingly, the remaining 35 participants were divided into two subgroups: 20 participants completed the OA and CPM assessments outside the scanner, while 15 participants underwent only the fMRI scanning session. In total, 25 participants completed both OA and CPM testing, and their data are included in the current analysis.

### Participants

Initially, twenty-five female FMS patients were enrolled in the study. Inclusion criteria required an FMS diagnosis at least three months before enrollment, following the American College of Rheumatology Fibromyalgia Criteria (ACR 16). Eligible participants were females aged 20–55, experiencing moderate to severe pain, defined as a score of ≥ 50 on the pain visual analog scale (VAS), with no history of severe psychiatric or neurological disorders, no serious systemic illnesses, and no history of alcohol or drug abuse. FMS patients were instructed to refrain from using SSRIs, SNRIs, GABA agonists, and opioid drugs for 48 h before the experiment Patients signed a declaration confirming they had not used cannabis in the two weeks prior to the experiment and agreed to abstain from cannabis throughout the entire study period. Exclusion criteria included acute pain, additional chronic pain conditions, rheumatic diseases, or regular cannabis use. The inclusion of only female participants in the study was decided to minimize bias, given that the majority of FMS patients are female (Shaver 2004). Additionally, the symptom profiles of FMS differ between men and women, further supporting the rationale for focusing the study population on females to ensure the consistency and relevance of the findings (Ruschak et al. 2023).

### Questionnaires

One to two days prior to the experimental session, FMS patients were asked to complete an online questionnaire assessing their medical history, along with the Twelve-Item Short Form of the McGill Pain Questionnaire (Melzack 1975), which was administered both at baseline and following the intervention in each session. This questionnaire captures the multidimensional nature of pain by evaluating its quality and intensity using 12 descriptors (e.g., throbbing, stabbing, aching), each rated on a numerical scale. It encompasses both sensory and affective dimensions to provide a comprehensive assessment of the patient’s pain experience.

### Study procedure

**Fig. 1:**
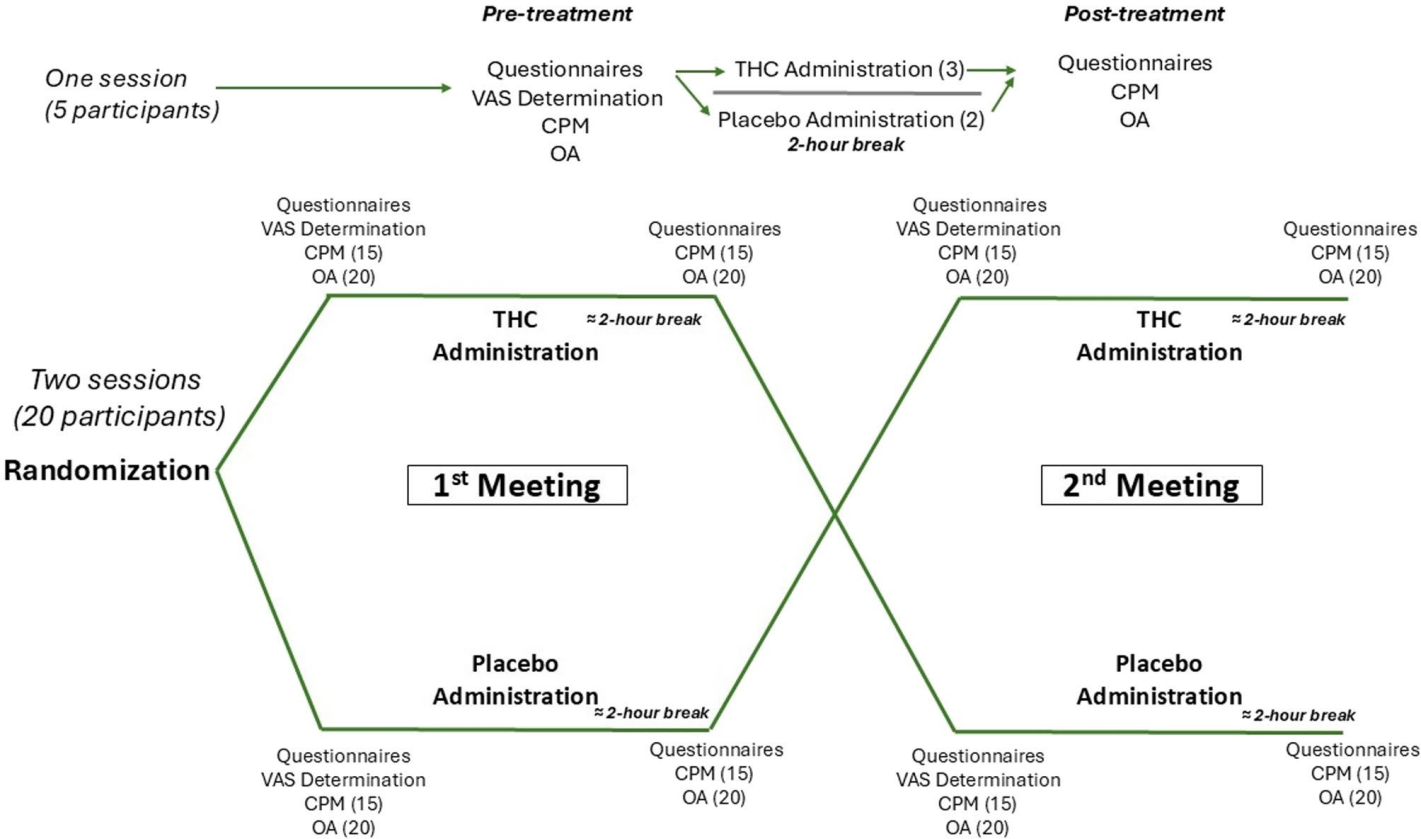
Illustrative paradigm of the study design, showing drug crossover randomization (THC vs. placebo) and pain assessment procedures. Participants who completed both experimental sessions underwent the McGill questionnaire (*N* = 20), VAS60 test (*N* = 20), CPM (*N* = 15), and OA (*N* = 20) at baseline, followed by the intervention. Each intervention consisted of either placebo or THC, administered in a counterbalanced, double-blind, randomized design. After a two-hour rest period, participants repeated the McGill questionnaire, CPM, and OA. Intervention sessions were separated by 2–6 weeks

The experiment consisted of two study sessions. Initially, a rheumatologist or pain specialist evaluated the selected FMS patients, following the previously outlined criteria. After a thorough explanation of the experimental procedure, all participants provided informed consent. At baseline, they completed the McGill questionnaire and underwent the VAS60 calibration test. Among the twenty-three patients selected, three completed only the THC session, undergoing the McGill questionnaire, OA, and CPM in a randomized order both before and after acute THC administration. A 5-minute interval was implemented between all sensory tests (VAS60 calibration, CPM, and OA) to minimize potential sensory habituation. Each session’s intervention consisted of either a placebo or THC, administered in a counterbalanced, double-blind, randomized manner. Randomization was performed by one of the blinded researchers affiliated with the study using the Excel RandBetween function, assigning “1” to placebo and “2” to THC. After a two-hour rest period, participants repeated the McGill questionnaire. Each intervention session was spaced between two to six weeks apart. The experimental design of the study is elucidated in Fig. 1.

### Treatment

In a double-blinded, placebo-controlled, cross-over design, patients were administered sublingual drops of THC or placebo oil at a dosage of 0.2 mg per kilogram. The THC oil contained 10% THC and 2% CBD, provided by Panaxia Pharmaceutical Industries, Lod, Israel. The placebo contained hemp oil with non-cannabinoids or psychoactive components. The dosage was established in accordance with a previous study that administered THC for radicular neuropathy (Weizman 2018). Of the twenty participants who completed both sessions, nine received THC during the first session, while eleven received the placebo in the first session.

**Fig. 2:**
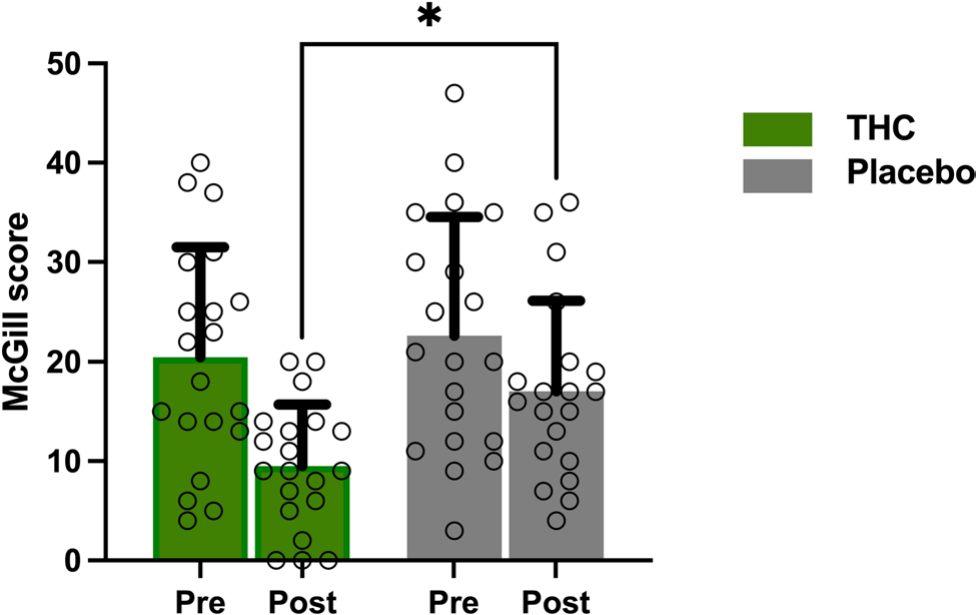
Effect of THC versus placebo on the McGill score (N = 20). THC significantly reduced pain ratings compared to placebo F(1,54) = 6.83, P = 0.012, partial η² = 0.11. No significant main effects of time (Pre vs Post, P=0.396) or treatment order (THC-first vs placebo-first, P=0.917) was found. Error bars represent the standard error of the mean (SEM)

### Individual calibration of pain assessments

Prior to the individual calibration procedure, participants completed a brief training session to familiarize themselves with the equipment and the sensations involved. They then underwent determination of the Visual Analogue Scale 60 (VAS60). The VAS60 score represents the temperature that elicits a pain rating of 60 on a pain scale ranging from 0 to 100. During the test, participants were instructed to focus on their sensations, differentiating between non-painful thermal sensations and painful ones. A visual, colored VAS scale was provided to all participants to aid in giving precise pain ratings for this test and all subsequent sensory pain paradigms in the study. Each participant was exposed to a series of thermal stimuli of varying temperatures on the volar side of their dominant arm, using a levels method (Medoc Main Station). They were asked to rate pain intensity immediately after each stimulus, using a numeric scale from 0 (no pain) to 100 (worst imaginable pain). The baseline temperature was set at 32 °C, with each stimulus lasting 7 s, increasing or decreasing by 5 °C per second, and with intervals between stimuli ranging randomly from 4 to 7 s. Between each stimulus, the temperature returned to the baseline of 32 °C, allowing participants to rate their perceived pain intensity. Notably, VAS60 is commonly applied in pain assessments like CPM and OA in a phasic manner. The 7-second duration provides enough time to induce a pain sensation and allows participants to rate it accurately. In repetitive-phasic stimulation, each stimulus is perceived as a separate unit, leading to a more defined pain sensation compared to tonic stimuli (Nahman-Averbuch et al. 2014; Yarnitsky et al. 2012; Granot et al. 2006). The test included nine trials with randomly ordered temperature intensities ranging between 37 °C and 47 °C. To prevent sensory habituation, the thermode was slightly repositioned after every three trials. Each participant’s VAS60 temperature was determined through simple linear regression, based on their pain ratings.

Thermal stimulation for all pain paradigms was delivered with a Peltier-based contact thermode (TSA II; Medoc Ltd, Ramat-Ishay, Israel) with a 30 mm × 30 mm contact probe. In each sensory test, the thermode was moved slightly on the volar forearm between repetitions to minimize habituation.

### Conditioned pain modulation (CPM)

The CPM assessment used a parallel paradigm, where two identical noxious “test stimuli” were applied: one before and one simultaneously with a noxious “conditioning stimulus” (Granot et al. 2008; Yarnitsky et al. 2008; Nirl et al. 2011; M 2008).

To evaluate pain caused by the test stimulus (TS_alone), each participant received a 30-second tonic heat stimulus on the volar side of their dominant arm at a temperature pre-determined to induce a VAS60 pain sensation, individualized for each participant. The baseline temperature was set at 32 °C, with a 2 °C per second increase and decrease rate. Participants rated pain intensity on a scale from 0 to 100 at three points during the stimulus: 0, 15, and 30 s. After a 15-minute break, participants immersed their non-dominant hand in a cold-water bath (up to the wrist) for 60 s. During the last 30 s of immersion, the “test stimulus” was reapplied, and participants provided pain intensity ratings once more. The CPM effect size was calculated by subtracting the average pain ratings from the test stimulus alone (TS_alone) from those recorded when the test stimulus was applied along with the conditioning stimulus (TS_conditioned). Negative values represented effective CPM.

The cold-water bath temperature ranged from 9 to 12 °C and was intended to elicit a mild to moderate pain rating of over 20 on a 0–100scale (Yarnitsky et al. 2015).

### Offset analgesia (OA)

The OA test followed a three-temperature paradigm applied to the volar aspect of the dominant arm (Ligato et al. 2018; Szikszay et al. 2021). Starting from a baseline temperature of 32 °C, the heat probe’s temperature increased at a rate of 2 °C per second until it reached each participant’s target test temperature, where it was held steady for 5 s. The temperature then rose by an additional 1 °C, remaining at this new level for another 5 s, before dropping back down by 1 °C to the participant’s original test temperature. This temperature was held for 20 s before returning to baseline at a rate of 5 °C per second. In this setup, the initial temperature increase reaching VAS 60 was labeled T1, the 1 °C increase held for 5 s was labeled T2, and the return phase to the VAS 60 temperature, maintained for 20 s, was labeled T3. Participants sequentially rated the intensity of the perceived pain with their non-dominant hand using a computerized Visual Analog Scale (COVAS) device. The magnitude of OA was computed by subtracting the peak COVAS rating T2 from the minimum COVAS rating within the initial 10 s of T3. Negative OA magnitude indicates efficient OA. The OA index was calculated to represent the success rate of OA, using the peak rating of T2 instead of just the change in COVAS. This calculation involved dividing the absolute OA magnitude by the T2 peak COVAS rating and then multiplying by 100 to express it as a percentage (Kobinata et al. 2017).

The OA test consisted of three trials, with a minimum 5-minute interval between each trial to prevent habituation. The thermode was slightly repositioned between trials to further reduce sensory habituation. The final OA magnitude was determined by averaging the results across the three trials, excluding any outliers. Outliers were identified through Z-scores, with values exceeding ± 3 excluded from the analysis (Oudejans et al. 2015; Ligato et al. 2018; Szikszay et al. 2021).

### Statistical analysis

We conducted the statistical analyses using GraphPad Prism version 9.5.0 for Windows (San Diego, CA, www.graphpad.com) and IBM SPSS Statistics (version v31, IBM Corp., Armonk, NY).

The sample size was determined using a power analysis conducted with G*Power software (version 3.1.9.7). The goal of the analysis was to ensure adequate statistical power to detect the effect of THC on CPM and OA compared to placebo, utilizing repeated measures ANOVA. The settings included one group, three measurements, a correlation among repeated measures of 0.5, and a non-sphericity correction of 1. The effect size was set at 0.5 (Yanes et al. 2019), with power and alpha levels established at 0.8 and 0.05, respectively. The analysis indicated that a minimum sample size of 17 participants was required. Given that the initial total number of participants was fixed at 40, we conducted a power analysis to confirm the minimum required sample size per group under this constraint. The analysis confirmed that dividing the sample evenly into two groups of 20 participants each would retain sufficient power to detect effects of moderate magnitude.

To examine treatment effects while accounting for treatment order, we conducted a 2 × 2 mixed-model analysis of variance (ANOVA) for all comparisons (McGill score \ CPM magnitude \ OA magnitude) with Treatment (THC vs. placebo) as a within-subject factor and Treatment Order (THC-first vs. placebo-first) as a between-subject factor. Time (Pre vs. Post) was included as an additional within-subject factor to evaluate changes relative to baseline. The model therefore tested for: (i) the main effects of Treatment, Time, and Order; (ii) the two-way interactions (Treatment × Time, Treatment × Order, Time × Order); and (iii) the three-way interaction (Treatment × Time × Order).

A mixed-model approach with subject as a random effect was used to account for repeated measures. Where appropriate, pairwise comparisons (pre vs. post within each treatment condition) were performed as follow-up analyses, with p-values adjusted for multiple testing using Bonferroni corrections. Effect sizes are reported as partial η².

Additionally, we utilized Pearson correlation to investigate the relationship between clinical measures and both tests as the Shapiro-Wilk test, which was used to assess normality, yielded non-significant results for all datasets.

All analyses were conducted using complete case data. Participants with missing values for any of the variables included in a given analysis were excluded from that specific analysis. No imputation methods were applied.

In all analyses, a significance level 0.05 was used to determine statistical significance. Both parametric and non-parametric tests were conducted with two-tailed considerations.

## Results

### General characteristics

Of the 25 participants initially enrolled, three withdrew after their first session with THC due to non-severe but intolerable side effects, and two withdrew after receiving placebo in their first session, citing personal reasons. Ultimately, 23 participants who received THC (mean age 40.93 ± 10.09 years) remained in the study. Among these, 20 participants completed both experimental sessions (THC and placebo). Of the twenty patients who completed both sessions, fifteen completed both the CPM and OA paradigms at each session, both pre-and post-intervention, while the remaining five only completed the OA test due to time constraints (twenty participants completed the OA task and fifteen completed the CPM task across both sessions).

The mean weight of the 23 participants was 65.38 kg (± 15.04), with an average height of 163.8 cm (± 6.10). The mean BMI was 24.44 (± 6.29). The average time since diagnosis was 6.15 (± 5.80) years. The average THC dose administered was 13.08 mg (± 3.00). The change in McGill score from baseline to post-THC administration was − 11.78 (± 8.72). None of the participants were regular cannabis users. The frequency of the most common side effects after THC treatment is listed in Table 1.

Side effects were gathered using a closed-ended questionnaire featuring potential side effects as outlined by Panaxia, the company from which the THC oil was purchased. No significant side effects were reported following the placebo treatment.

**Table 1:** Frequency of the most common side effects: side effects reported among participants who received THC (*N* = 23). Frequency is defined as the number of patients experiencing a specific side effect divided by the total number of participants

Side effect	Frequency (%)
Drowsiness	21.7
Dry mouth	21.7
Anxiety	17.3
Ataxia	8.6
Confusion	26.08
Decreased concentration	52.17
Disassociation	13.04
Orthostatic hypotension	4.34
Nausea	56.52
Headache	34.78

### Effect of THC on spontaneous pain rating

To assess the effect of THC on spontaneous pain ratings (McGill scores), a 2 × 2 mixed-design ANOVA was conducted with Treatment (THC vs. placebo) and Time (Pre vs. Post) as within-subject factors, and Order (THC-first vs. placebo-first) as a between-subject factor.

 The analysis revealed a significant main effect of Treatment, F(1, 54) = 6.83, P = 0.012, partial η² = 0.11, with lower pain ratings in the THC condition compared with placebo. The main effect of Time was not significant, F(1, 54) = 0.73, P = 0.396, partial η² = 0.01, nor was the main effect of Order, F(1, 54) = 0.01, P = 0.917, partial η² < 0.01.

 None of the interaction terms reached significance: Treatment × Time, F(1, 54) = 0.45, P = 0.503, partial η² = 0.01; Treatment × Order, F(1, 54) = 1.52, P = 0.223, partial η² = 0.03; Time × Order, F(1, 54) = 0.94, P = 0.337, partial η² = 0.02; and the Treatment × Time × Order interaction, F(1, 54) = 0.005, P = 0.945, partial η² < 0.001. Mean pain ratings in the baseline, THC and placebo conditions are shown in Table 2.

**Table 2:** Psychophysical and clinical measurements at baseline (pretreatment) and after placebo and THC treatments. The values are presented as the means and (SD). Pain ratings were recorded on a scale ranging from 0 to 100

Psychophysical and clinical measurements				
	Pre-THC	THC	Pre-placebo	Placebo
McGill score	20.45 (11.06)	9.5 (6.21)	22.65 (11.89)	17.05 (9.05)
∆ McGill		−11.05 (9.42)		−5.6 (13.45)
Mean VAS60 temperature (°C)	43.92 (2.99)		44.2 (1.69)	
Mean pain rating during test stimulus (TS_alone)	62.34 (28.61)	51.65 (25.72)	60.86 (33.90)	49.72 (32.5)
Mean pain rating during CPM (Ts_Conditioned)	67.31 (17.21)	60.22 (32.82)	64.54 (11.34)	55.10 (9.38)
∆ CPM magnitude		−3.36 (19.42)		0.96(23.5)
Peak pain rating at T2 temperature during OA	55.58 (14.32)	69.23 (22.26)	65.68 (16.57)	62.75 (21.47)
Minimum pain rating during T3 temperature (first10 seconds) during OA	28.16 (25.7)	12.85 (33.58)	28.58 (20.68)	23.08 (26.87)
∆ OA magnitude		−21.43 (32.94)		−3.67 (26.46)

### Effect of THC on OA

**Fig. 3:**
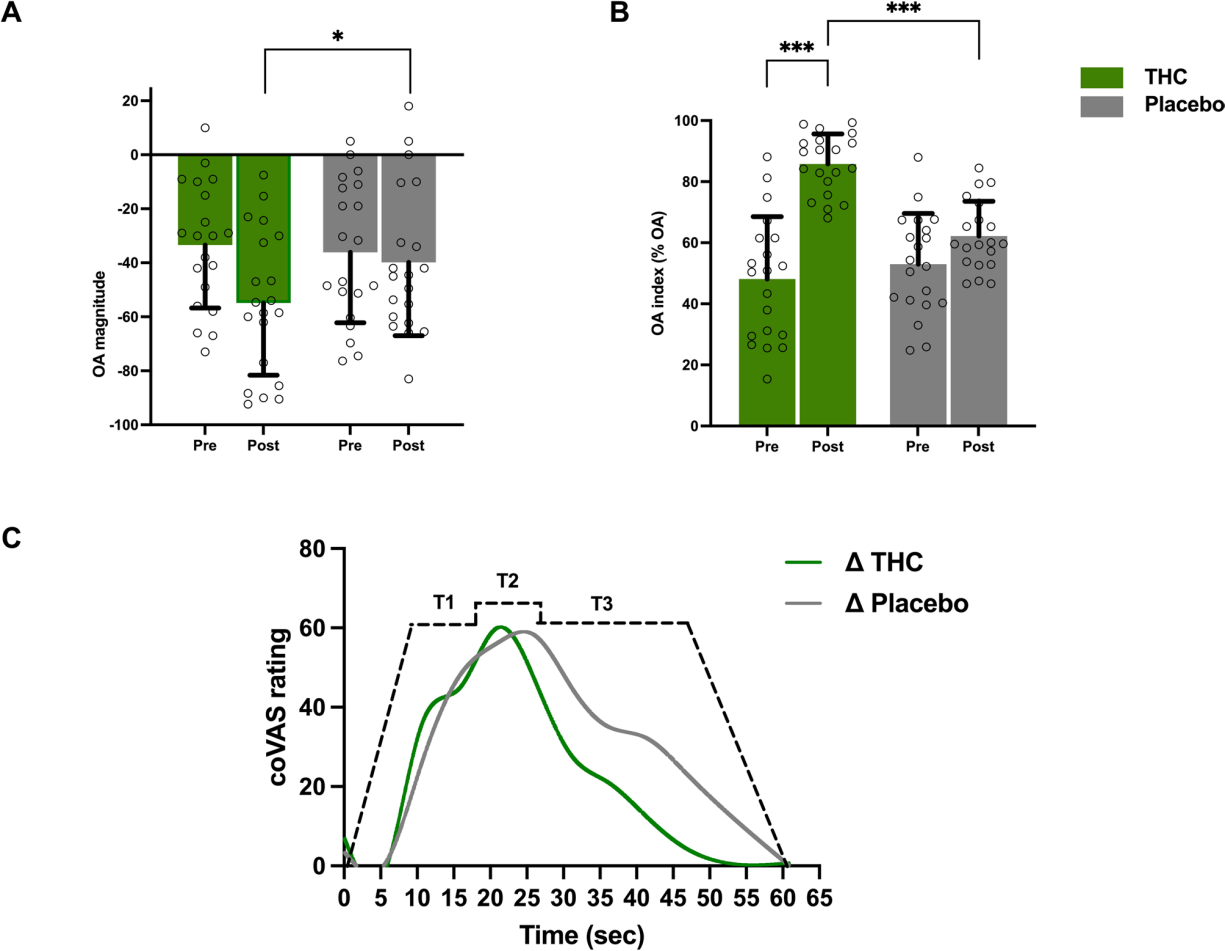
Effect of THC on OA magnitude and OA index (*N* = 20). **A** THC administration significantly enhanced OA magnitude, reflected in a significant main effect of Treatment, F(1, 54) = 5.85, *p* = 0.020, partial η² = 0.10. Negative values indicate stronger OA responses. **B** OA index (%) at baseline compared with THC and placebo. The OA index was significantly higher following THC relative to placebo, as shown by a significant main effect of Treatment, F(1, 54) = 17.34, *p* < 0.001, partial η² = 0.24. In addition, a significant Treatment × Time interaction was observed, F(1, 54) = 8.99, *p* = 0.004, partial η² = 0.14, indicating that the pre-to-post increase was greater in the THC condition than in placebo. **C** Representative trace of continuous pain ratings during OA (0–100), averaged across three trials. “THC” values represent the average of THC and baseline, while “PLC” values represent the average of placebo and baseline. Error bars represent the standard error of the mean (SEM)

 To assess the effect of THC on OA, a 2 × 2 mixed-design ANOVA was conducted with Treatment (THC vs. placebo) and Time (Pre vs. Post) as within-subject factors, and Order (THC-first vs. placebo-first) as a between-subject factor. The analysis revealed a significant main effect of Treatment, F(1, 54) = 5.85, p = 0.020, partial η² = 0.10, indicating that OA responses were overall lower in the THC condition compared with placebo. In contrast, the main effect of Time was not significant, F(1, 54) = 0.12, p = 0.73, partial η² < 0.01, nor was the main effect of Order, F(1, 54) = 1.45, p = 0.23, partial η² = 0.03. None of the interaction terms reached significance: Treatment × Time, F(1, 54) = 1.02, p = 0.32, partial η² = 0.02; Treatment × Order, F(1, 54) = 0.41, p = 0.53, partial η² = 0.01; Time × Order, F(1, 54) = 0.58, p = 0.45, partial η² = 0.01; and the three-way interaction Treatment × Time × Order, F(1, 54) = 0.23, p = 0.63, partial η² < 0.01. These findings suggest that THC exerted a significant effect on OA, independent of timepoint or treatment order, and that no higher-order interactions were present (Figs. 3A and C).

 To further evaluate the efficacy of OA, we analyzed the OA index, which quantifies the magnitude of OA on a scale from 0 to 100 (Fig. 3B). A 2 × 2 mixed-design ANOVA with the same parameters described above was performed. The analysis revealed a significant main effect of Treatment, F(1, 54) = 17.34, p < 0.001, partial η² = 0.24, with higher OA values in the THC condition compared with placebo. The main effect of Time was not significant, F(1, 54) = 0.05, p = 0.828, partial η² = 0.001, and the main effect of Order was also not significant, F(1, 54) = 1.91, p = 0.173, partial η² = 0.03. A significant Treatment × Time interaction emerged, F(1, 54) = 8.99, p = 0.004, partial η² = 0.14, indicating that the pre-to-post increase in OA index was greater following THC than placebo. The Treatment × Order interaction was not significant, F(1, 54) = 2.34, p = 0.132, partial η² = 0.04, nor was the Time × Order interaction, F(1, 54) = 2.86, p = 0.097, partial η² = 0.05. Finally, the three-way Treatment × Time × Order interaction was not significant, F(1, 54) = 0.38, p = 0.541, partial η² = 0.007. Mean pain ratings of all participants during the OA paradigm is presented in Table 2.

### Effect of THC on CPM

**Fig. 4:**
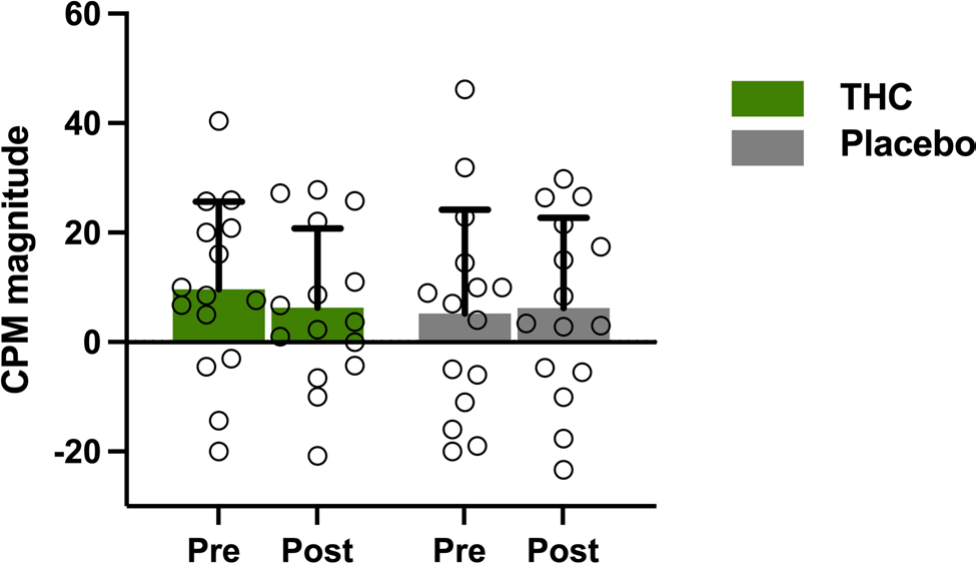
Effect of THC on CPM magnitude (*N* = 15). THC administration did not significantly affect CPM magnitude, as reflected by a non-significant main effect in the 2 × 2 mixed-design ANOVA (F(1, 39) = 0.20, *P* = 0.658, partial η² = 0.005)

CPM was experimentally valid in fifteen participants (*N* = 15). THC administration did not significantly alter CPM magnitude, as indicated by a 2 × 2 mixed-design ANOVA conducted with the same parameters described previously. The main effect of Treatment was not significant, F(1, 39) = 0.20, *p* = 0.658, partial η² = 0.005. Similarly, the main effects of Time (F(1, 39) = 0.51, *p* = 0.479, partial η² = 0.013) and Order (F(1, 39) = 0.57, *p* = 0.455, partial η² = 0.014) were not significant. No significant interactions were observed: Treatment × Time (F(1, 39) = 0.03, *p* = 0.868, partial η² = 0.001), Treatment × Order (F(1, 39) = 0.41, *p* = 0.527, partial η² = 0.010), or Time × Order (F(1, 39) = 0.04, *p* = 0.838, partial η² = 0.001). The three-way Treatment × Time × Order interaction was also not significant (F(1, 39) = 0.24, *p* = 0.627, partial η² = 0.006). These results are illustrated in Fig. 4, and the mean pain ratings across participants during the CPM paradigm are presented in Table 2.

### Baseline OA predicts the efficacy of THC-dependent pain reduction

A total of twenty-three participants were included in the OA correlation analysis (including three participants who completed only the THC session), while 18 participants were analyzed for the CPM correlation (including three who completed only the THC session).

**Fig. 5:**
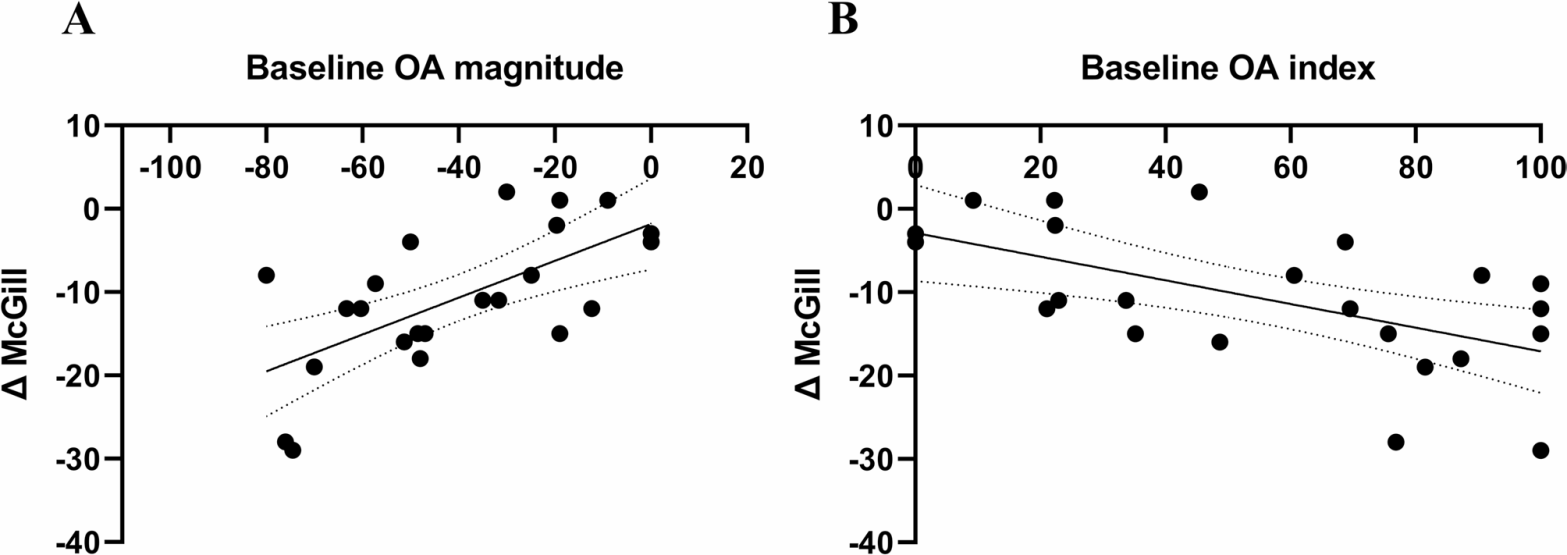
Predictive efficacy of baseline OA on pain reduction following THC treatment. **A** Displays a significant linear correlation between baseline OA magnitude and the change in ΔMcGill scores (post-THC – pre-THC), showing a positive correlation (R² = 0.404, *P* = 0.003). **B** Similarly, a significant correlation was observed between baseline OA index and ΔMcGill scores (R² = 0.342, *P* = 0.003). These results indicate that a higher baseline OA magnitude and OA index were predictive of more significant pain reduction following THC treatment (*N* = 23)

To evaluate whether baseline OA magnitude could predict the degree of analgesia provided by THC, we calculated the change in McGill scores from baseline to post-THC (ΔMcGill_THC_). Simple linear regression analysis was performed using baseline OA magnitude and OA index values as predictors (*N* = 23). A significant linear relationship was found between baseline OA magnitude and ΔMcGill scores (R² = 0.404, F(1, 21) = 11.54, *P* = 0.003), as illustrated in Fig. 5A. Similarly, the OA index showed a significant correlation with ΔMcGill scores, with higher OA index values corresponding to greater pain reduction (R² = 0.342, F(1, 21) = 10.94, *P* = 0.003), as seen in Fig. 5B.

**Fig. 6:**
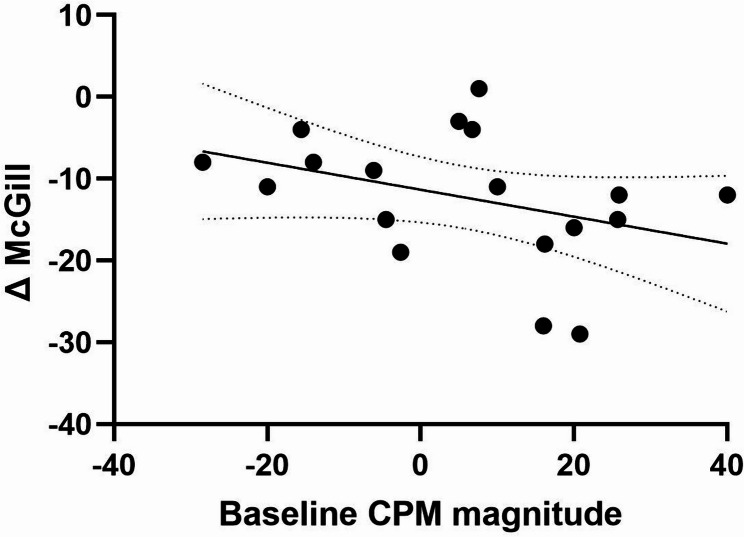
Predictive efficacy of baseline CPM magnitude on pain reduction following THC treatment. The figure illustrates the negative correlation between baseline CPM magnitude and the reduction in pain ratings (ΔMcGill) following THC treatment. The correlation was not statistically significant (Rp(18) = −0.357, *P* = 0.121)

To examine whether the prediction of treatment response was specific to THC after accounting for placebo effects, we tested the correlations between baseline OA magnitude and (i) the change in McGill scores from baseline to post-placebo (ΔMcGill_placebo_) among the 20 participants who completed both experimental sessions, and (ii) the net effect (ΔMcGill_**THC**_ – ΔMcGill_placebo_). Both analyses yielded non-significant results: the correlation between baseline OA magnitude and ΔMcGill_placebo_ was not significant (Rp(20) = 0.366, *p* = 0.123, 95% CI [–0.105, 0.703]), and likewise, the correlation with the net effect (ΔMcGill_THC_ – ΔMcGill_placebo_) was not significant (Rp(20) = 0.01, *p* = 0.965, 95% CI [–0.445, 0.462]). Moreover, the correlation between baseline CPM magnitude values and ΔMcGill scores was non-significant (Rp(18) = −0.357, *P* = 0.121, 95% CI [−0.69, 0.10]) as shown in Fig. 6.

## Discussion

In this double-blind, placebo-controlled randomized clinical trial a single dose administration of THC-rich oil produced clinical analgesia and enhancement of OA, a measure of central pain modulation, in patients with FMS. Interestingly, CPM, a different measure of descending pain inhibition, was not affected by THC. Moreover, we found that the baseline magnitude of individual OA responses, but not those of the CPM, was positively correlated with the degree of pain relief experienced following THC treatment in patients.

The reduction in spontaneous pain ratings following THC administration compared to placebo, as indicated by the short-form McGill score, (Fig. 2), is consistent with previous clinical studies suggesting that cannabinoids, including THC, can reduce pain intensity and improve quality of life in patients with central pain syndromes including those with FMS (Safi et al. 2024; Weber et al. 2009; McDonagh et al. 2022). Furthermore, a single low dose of THC has been found to produce analgesic effects in patients with chronic radicular neuropathy, linked to decreased functional connectivity in the ACC and sensorimotor cortex, which play roles in cognitive-emotional modulation (Weizman 2018).

Cannabinoid-based treatments have been proposed for nociplastic pain, which is typically of central origin and characterized by nervous system sensitization without a clear physiological cause to account for the severity of symptoms (Bułdyś et al. 2023). Cannabis-based medicines, particularly those containing THC, have demonstrated potential benefits in several clinical trials and observational studies where patients commonly report improvements in pain, sleep, and mood (Fitzcharles et al. 2021). These findings suggest that cannabinoids may serve as a promising adjunctive therapy for nociplastic pain, though further rigorous research is needed to establish their efficacy and safety.

Interestingly, we observed a notable improvement in OA index and magnitude post-THC administration compared to the initial baseline as well as placebo (Fig. 3), however, this effect was not observed for CPM. OA has been shown to trigger activation in cortical brain regions in healthy individuals, including the anterior insula, dorsolateral prefrontal cortex (dlPFC), mid-cingulate cortex (MCC), and the ACC, but also in brainstem pain-modulating areas such as the RVM and PAG (Alter et al. 2022; Li et al. 2022; Derbyshire and Osborn 2009; Zhang et al. 2018). The specific impact of THC on OA might be linked to findings from another study (Weizman 2018), that observed a THC-induced decrease in the functional connectivity of the dlPFC, which is notably active during OA, and other brain regions involved in central pain processing, such as the ACC (Weizman 2018)and brainstem areas (Weizman et al. 2024). This may be attributed to enhanced pain modulatory functions of the ACC and dlPFC following THC administration, which could have facilitated the observed improvement in the OA response.

Notably, in this study, THC did not influence CPM magnitude, which is linked to substantial involvement of core brainstem areas such as the PAG and RVM (Nahman-Averbuch and Timmers 2023), with some contribution of higher emotional-cognitive cortical areas such as the ACC, dlPFC and insula (Ossipov et al. 2010). It is possible that the well-known dysfunctions in these cortical circuits in FMS patients may limit the impact of THC on CPM. It may also be that the modulatory effects of THC on these cortical areas preferentially affect their interactions with the brainstem, which is highly involved in CPM, thus attenuating the potential effects of THC on CPM magnitude. Notably, a previous study with a small sample size (Weizman et al. 2024)reported an enhancement in CPM following THC administration for neuropathic pain, whereas another study that assessed CPM after administration of the cannabinoid Nabilone had no effect on healthy volunteers (John Redmond et al. 2008). This finding could imply that the impact of THC on mechanisms of pain modulation in chronic pain conditions might be disease specific.

This divergence in central mechanisms between the two paradigms underscores the greater reliance of OA on cortical-driven pain modulation compared to CPM, reinforcing their distinct roles in spatial and temporal processing of nociceptive input. These differences may help explain the differential effects of THC observed across the two pain modulation paradigms.

Another notable result was that baseline OA magnitude predicted the capacity for THC analgesia from baseline, as determined by the McGill questionnaire (Fig. 5). Patients exhibiting stronger initial OA displayed a larger reduction in their pain ratings after THC treatment, suggesting that a better rapid pain modulation at baseline may signal greater adaptability in pain modulatory patterns in FMS. The clinical significance of this finding is that a simple and quick test like OA could potentially help identify which patients are more likely to benefit from THC treatment, paving the way for personalized pain management.

However, when we examined whether this predictive effect was specific to THC after controlling for placebo responses, no significant correlations emerged. Baseline OA magnitude did not predict changes from baseline in McGill scores in the placebo condition, nor did it predict the net THC effect (ΔTHC – Δplacebo). This pattern indicates that OA is associated with the observed clinical response in the THC arm but does not appear to exclusively capture the isolated pharmacological component of THC analgesia once placebo-related effects are subtracted. Given the strong and variable placebo responses in FMS, it is possible that mathematically removing placebo also eliminates clinically meaningful variance that OA reflects. Taken together, our findings suggest that OA may represent a general biomarker of central pain modulation and overall analgesic responsiveness, rather than a predictor restricted solely to the drug-specific effects of THC. To the best of our knowledge, this is the first study to suggest a potential clinical predictive role for OA in tailoring analgesic treatment.

While prior studies have demonstrated the predictive capabilities of different treatments, including SNRIs, on CPM (Yarnitsky et al. 2012; Sugimine et al. 2017), our research tested for the first time the correlation between baseline CPM magnitude and pain reduction following THC treatment. Although trends were evident in the association between CPM and treatment response, these did not reach statistical significance, highlighting the need for further investigation in larger cohorts.

Various pharmacological studies have indicated differences between CPM and OA responses. For example, in patients with diabetic polyneuropathy, opioid agonists increased CPM effectiveness (Niesters et al. 2014). In contrast, neither opioid agonists nor antagonists had any effect on OA in individuals experiencing opioid-induced hyperalgesia (Martucci et al. 2012). Furthermore, administering an SNRI enhances CPM in patients with diabetic neuropathies (Yarnitsky et al. 2012), whereas a drug from the same class does not affect OA in healthy individuals (Larsen et al. 2022). These variations may help to further clarify the distinctions between these central pain modulation paradigms, although the research remains inconclusive (France et al. 2016; Okkerse et al. 2017).

Remarkably, the differences between the mechanisms of CPM and OA seem to be maintained after pharmacological treatment, as seen in recent research by De Vita and colleagues (De Vita et al. 2022) examining the effects of CBD on OA and CPM in healthy individuals. The study showed that CBD enhanced CPM in healthy individuals, while its influence on OA seemed primarily expectancy driven. This suggests that CBD may act predominantly through spinal or peripheral mechanisms with limited impact on OA, highlighting a distinct psychological-cognitive component underlying OA compared to CPM. These differences may further explain the divergent effects of THC

on the two paradigms. Accordingly, the fact that distinct mechanisms support CPM and OA is further highlighted by their differing correlation patterns with spontaneous pain ratings reductions following THC treatment in the current study, as a successful initial OA predicted a better response to treatment, while a less successful initial CPM correlated with a better treatment response. This may suggest that better cortical modulation mechanisms such as seen in OA facilitate THC analgesia, while, in turn, THC analgesia normalizes aberrant brainstem pain modulation as seen in maladaptive CPM.

## Conclusions

To conclude, this study corroborates the possible effectiveness of THC in alleviating experimental and spontaneous pain in FMS, a study case of central sensitization, and shows an enhancement of OA responses after THC treatment in FMS patients compared to baseline and placebo. This result was not replicated for CPM, further highlighting the distinct mechanisms both paradigms rely on. Moreover, we showed -for the first time- the potential of OA to predict THC induced analgesia. This, in turn, reinforces the potential of OA as a reliable marker of pain modulation in FMS and may pave the way for personalized cannabinoid-based therapies for chronic pain in the future. Further research is essential to deepen our understanding of the neural mechanisms by which THC influences pain modulation in central pain conditions. 

## Limitations

While the findings of this study are very interesting, some limitations should be acknowledged. First, we did not include a control condition in the OA paradigm involving a constant thermal stimulus matched for duration and intensity. The lack of this control may have resulted in an overestimation of OA responses, as the analysis did not account for potential baseline sensory adaptation or habituation effects. Moreover, the use of a psychoactive substance in a randomized, double-blinded trial presents certain challenges. Many participants experienced side effects after THC administration, which may have compromised the blinding, as they could have anticipated the treatment they received. Unfortunately, we did not ask participants at the end of each session to indicate which treatment they believed they had received, something we strongly recommend for future pharmacological studies of this nature. However, we examined whether there was an effect of drug administration order, and no such effect was found.

## Supplementary Information

Supplementary Material 1.

Supplementary Material 2.

Supplementary Material 3.

Supplementary Material 4.

Supplementary Material 5.

Supplementary Material 6.

Supplementary Material 7.

Supplementary Material 8.

Supplementary Material 9.

## Data Availability

The datasets generated and/or analysed during the current study are not publicly available due to privacy concerns in accordance with the Tel Aviv Sourasky Medical Center’s privacy policy but are available from the corresponding author on reasonable request.

## Abbreviations

FMS: Fibromyalgia
CPM: Conditioned pain modulation
OA: Offset analgesia
THC: Tetrahydrocannabinol
CBD: Cannabidiol
ACC: Anterior cingulate cortex
PAG: Periaqueductal grey
dlPFC: Dorsolateral prefrontal cortex
RVM: Rostral ventromedial medulla
