# Supplementary material for: The impact of tetrahydrocannabinol on central pain modulation in chronic pain: a randomized clinical comparative study of offset analgesia and conditioned pain modulation in fibromyalgia
Source: J Cannabis Res. 2025 Nov 6;7:86. doi: 10.1186/s42238-025-00348-x (PMC12590702; doi:10.1186/s42238-025-00348-x)
Supplement: Supplementary file 2 — Supplementary Material 2. [file 42238_2025_348_MOESM2_ESM.docx]

**Figure 1: CONSORT 2025 Flow Diagram**

Flow diagram of the progress through the phases of a randomised trial of two groups (that is, enrolment, intervention allocation, and data analysis)

Randomised (completed two sessions); n= 20

Analyzed (n=11)

Analyzed (n=9)

Analysis

Excluded (n= 5 )

Not meeting inclusion criteria (n=0)

Declined to participate (n=2 )

Other reasons (n= 3 )

Allocation

Allocated to intervention (n=9 )

Received THC at first session and placebo at second session

Allocated to intervention (n= 11 )

Received placebo at first session and THC at second session

Enrolment

Assessed for eligibility (n= 25 )
