# Supplementary figures and images for: The impact of tetrahydrocannabinol on central pain modulation in chronic pain: a randomized clinical comparative study of offset analgesia and conditioned pain modulation in fibromyalgia
Source: J Cannabis Res. 2025 Nov 6;7:86. doi: 10.1186/s42238-025-00348-x (PMC12590702; doi:10.1186/s42238-025-00348-x)

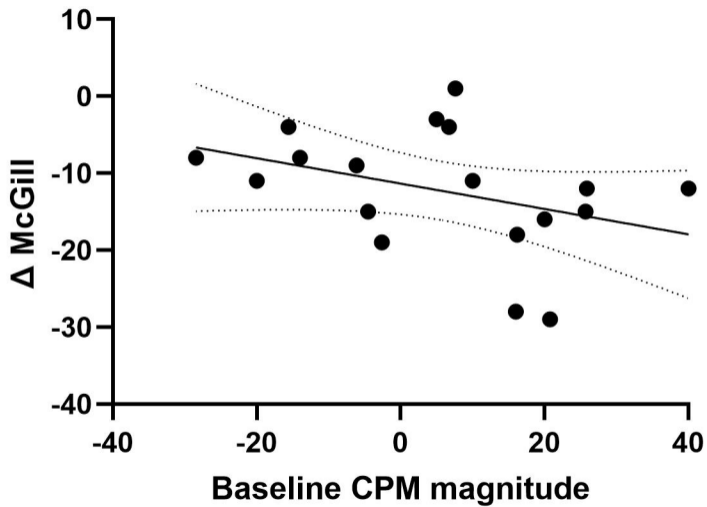

Supplement: Supplementary file 3 — Supplementary Material 3. [file 42238_2025_348_MOESM3_ESM.pdf]

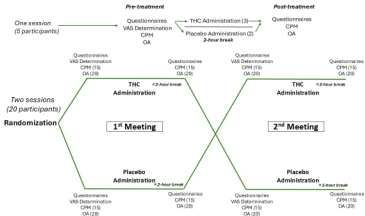

Supplement: Supplementary file 4 — Supplementary Material 4. [file 42238_2025_348_MOESM4_ESM.pdf]

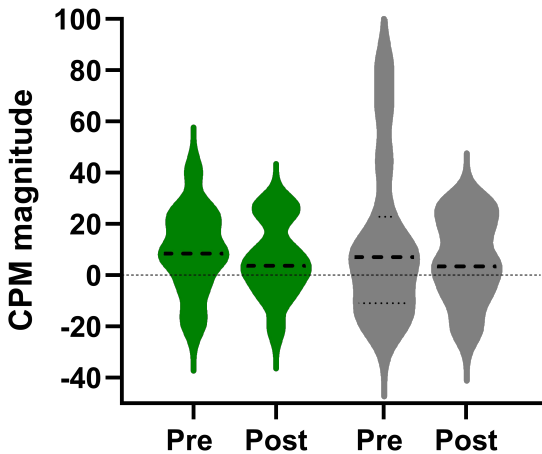

Supplement: Supplementary file 5 — Supplementary Material 5. [file 42238_2025_348_MOESM5_ESM.pdf]

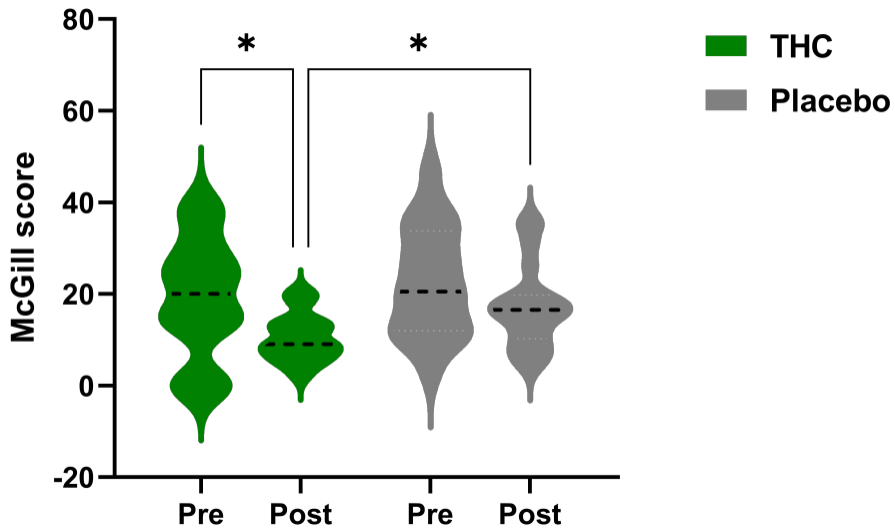

Supplement: Supplementary file 6 — Supplementary Material 6. [file 42238_2025_348_MOESM6_ESM.pdf]

**A****Baseline OA magnitude**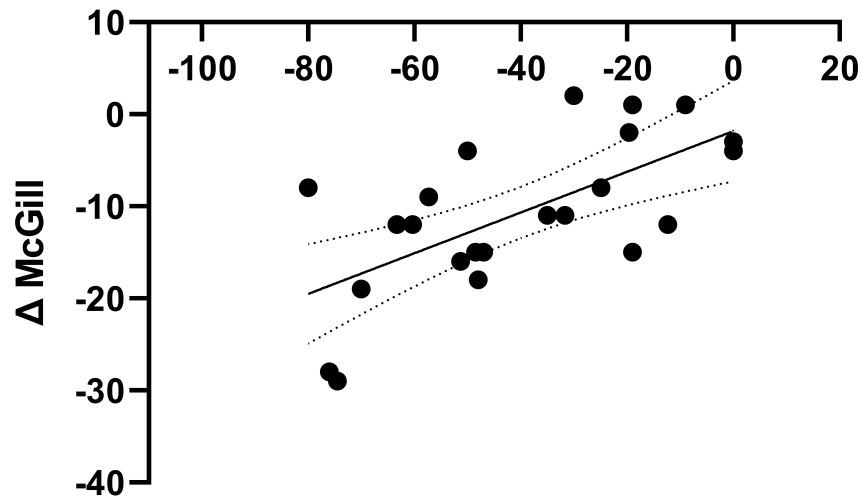**B****Baseline OA index**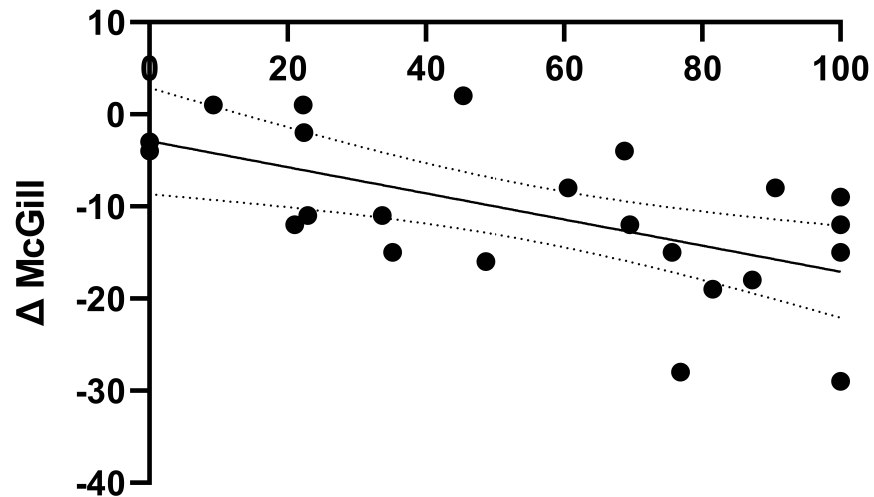

Supplement: Supplementary file 7 — Supplementary Material 7. [file 42238_2025_348_MOESM7_ESM.pdf]

**A**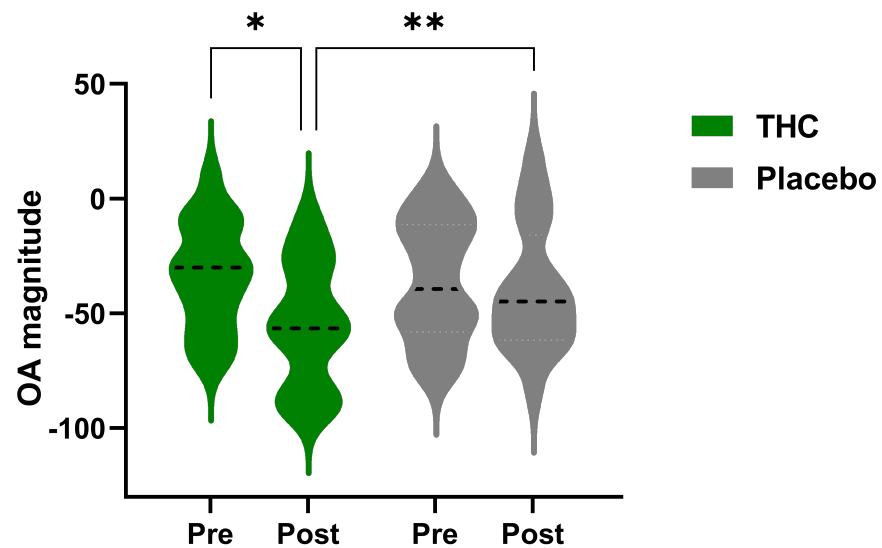**B**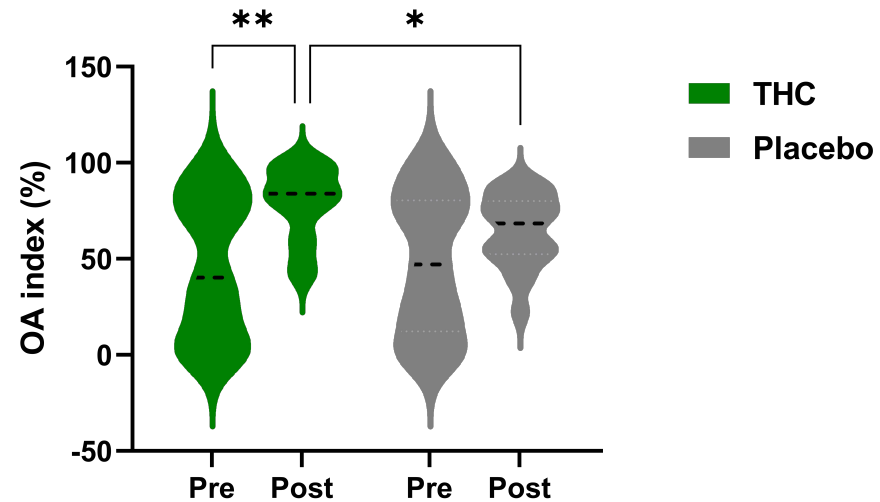**C**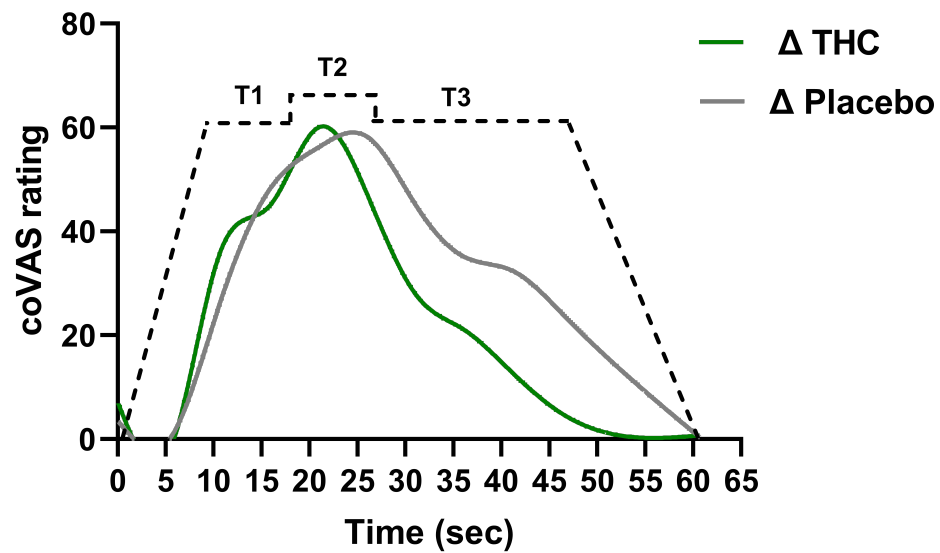

Supplement: Supplementary file 8 — Supplementary Material 8. [file 42238_2025_348_MOESM8_ESM.pdf]
