# Supplementary material for: The impact of tetrahydrocannabinol on central pain modulation in chronic pain: a randomized clinical comparative study of offset analgesia and conditioned pain modulation in fibromyalgia
Source: J Cannabis Res. 2025 Nov 6;7:86. doi: 10.1186/s42238-025-00348-x (PMC12590702; doi:10.1186/s42238-025-00348-x)
Supplement: Supplementary file 9 — Supplementary Material 9. [file 42238_2025_348_MOESM9_ESM.docx]

**Additional file 1: Individual data corresponding to the bar graphs presented in Figures 2, 3, and 4 of the main manuscript**


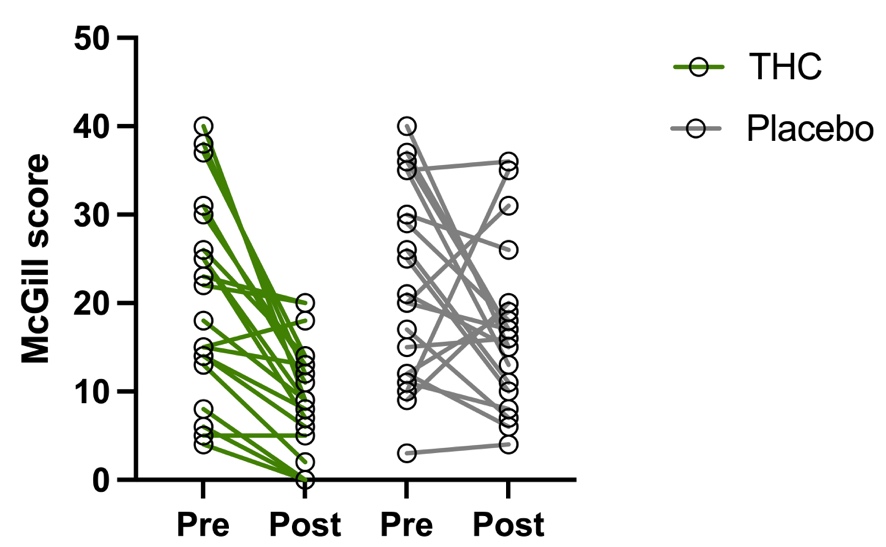


**Supp_Figure 1. Individual values of McGill scores in the THC and placebo conditions.** The graph displays individual values at baseline and after treatment for each experimental condition. McGill pain scores were **20.45 ± 11.06** at baseline and **9.50 ± 6.21** after THC administration. In the placebo condition, scores were **22.65 ± 11.89** at baseline and **17.05 ± 9.05**after placebo administration.


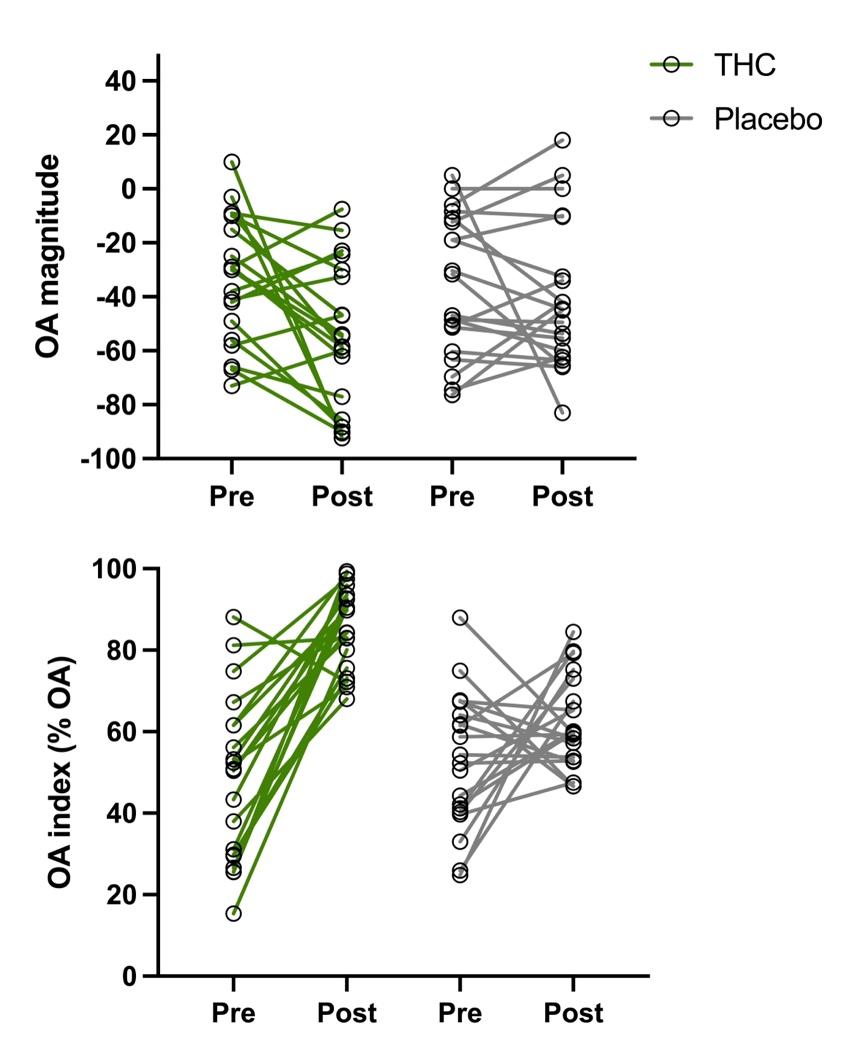


**Supp_Figure 2. Individual values of OA magnitude and OA index in THC and placebo conditions**. The graph displays individual values at baseline and after treatment for each experimental condition. For OA magnitude, the scores were –33.45 ± 23.27 at baseline and –54.88 ± 26.71 after THC administration. In the placebo condition, scores were –36.13 ± 26.07 at baseline and –39.80 ± 27.19 following placebo administration. For the OA index, values were 48.12 ± 20.40 at baseline and 85.78 ± 9.83 after THC administration. In the placebo condition, the OA index was 52.99 ± 16.63 at baseline and 62.20 ± 11.35 following placebo administration.


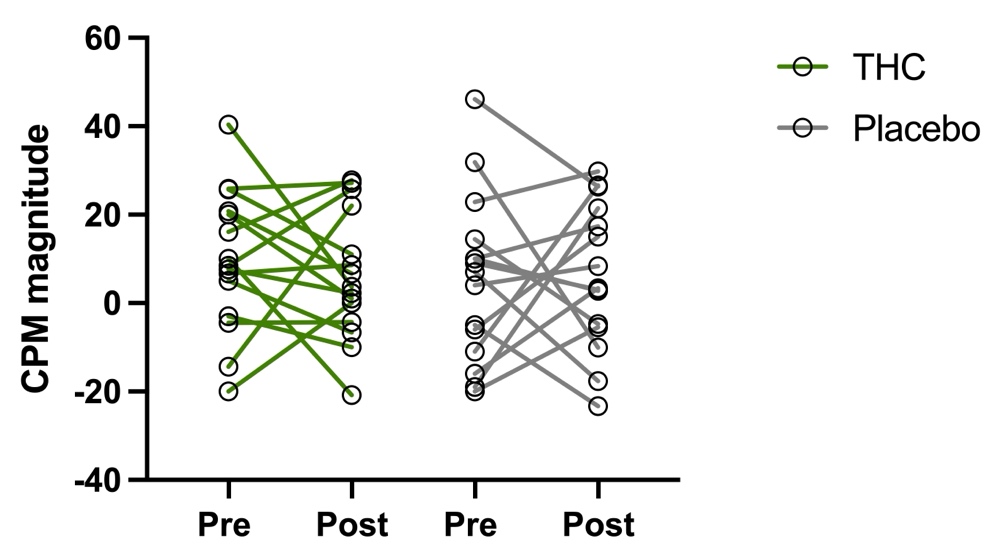


**Supp_Figure 3. Individual values of CPM magnitude in THC and placebo conditions**. The mean ± SD values for CPM magnitude were 9.65 ± 16.02 at baseline and 6.29 ± 14.45 after THC administration. In the placebo condition, scores were 5.22 ± 18.92 at baseline and 6.19 ± 16.49 following placebo administration.
